# Efficacy of cyclic and extended regimens of ethinylestradiol 0.02 mg ‐levonorgestrel 0.09 mg for dysmenorrhea: A placebo‐controlled, double‐blind, randomized trial

**DOI:** 10.1002/rmb2.12373

**Published:** 2021-02-27

**Authors:** Tasuku Harada, Mikio Momoeda

**Affiliations:** ^1^ Department of Obstetrics and Gynecology Tottori University Faculty of Medicine Yonago Japan; ^2^ Department of Integrated Women’s Health St. Luke’s International Hospital Tokyo Japan

**Keywords:** dysmenorrhea, ethinylestradiol, levonorgestrel, low‐dose estrogen/progestin combination product, placebo‐controlled randomized trial

## Abstract

**Purpose:**

We aimed to evaluate the efficacy and safety of 28‐day Cyclic and 84‐day Extended regimens of NPC‐16 (ethinylestradiol 0.02 mg plus levonorgestrel 0.09 mg) in patients with dysmenorrhea.

**Methods:**

This was a placebo‐controlled, double‐blind, randomized trial conducted in Japan. A total of 251 primary and secondary dysmenorrhea patients were randomly assigned to the NPC‐16‐Cyclic group, NPC‐16‐Extended group, or the Placebo group. The primary end point was a comparison of the efficacy and safety of the Cyclic and Extended NPC‐16 regimen for the treatment of dysmenorrhea relative to the Placebo.

**Main findings:**

Significantly greater reductions in total dysmenorrhea score and visual analog scale score were observed in the Cyclic and Extended groups compared with the Placebo group. Compared with the Cyclic regimen as a secondary end point, the Extended regimen exhibited greater efficacy in the treatment of dysmenorrhea over the course of the study period, particularly in patients with severe dysmenorrhea. The incidence of adverse drug reactions (ADRs) was significantly higher in the Cyclic and Extended groups than in the Placebo group.

**Conclusion:**

The Cyclic and Extended regimens of NPC‐16 significantly reduced dysmenorrhea severity compared to placebo. The Extended regimen was superior to cyclic regimen in reducing the dysmenorrhea.

## 1 INTRODUCTION

Dysmenorrhea is a common gynecologic problem, reportedly affecting almost half of menstruating women. Of those affected, approximately 5%‐15% experience pain of such severity that it limits their ability to work or study, resulting in periods of workplaces or school absence.^1^, ^2^ Dysmenorrhea is also a major symptom of endometriosis, which affects 10% of reproductive‐age women. Severe, persistent dysmenorrhea at the time of adolescence is a diagnostic marker of adult endometriosis.^3^ It was recently proposed that women with severe primary dysmenorrhea should receive early treatment with hormonal drugs before a definite diagnosis of endometriosis by surgical laparoscopy is made, as “look and treat” surgery does not always result in a favorable outcome in terms of the patient's endometriosis life.^4^ Such potentially unfavorable outcomes are due to the high recurrence rate after surgery and damage to the ovarian reserve after endometrioma surgery.^5^

The use of combined oral contraceptives (OCs) is beneficial for the treatment of dysmenorrhea. We previously conducted randomized controlled trials (RCTs) of the estrogen/progestin combination product IKH‐01 (ethinylestradiol [EE] 0.035 mg and norethisterone 1 mg) for the treatment of primary dysmenorrhea and endometriosis‐associated dysmenorrhea, and NPC‐01 (EE 0.02 mg and norethisterone 1 mg) for the treatment of dysmenorrhea.^6^, ^7^, ^8^ The results of these RCTs demonstrated that low‐dose OCs are efficacious for controlling pain in patients with either primary or secondary dysmenorrhea.

Compared with a 28‐day cyclic regimen, extended regimens significantly reduce the duration of menstrual pain in women using the contraceptives.^9^ In our recent RCT conducted in Japan, a low‐dose OC (EE 0.02 mg and drospirenone 3 mg) extended regimen (flexible) substantially alleviated both dysmenorrhea and also non‐menstrual pain as well as deep dyspareunia in endometriosis patients.^10^ Those findings were confirmed by data from a recent systematic‐review.^11^ Other researches have indicated that use of OCs for longer than 6 months can significantly reduce the size of ovarian endometriomas.^6^, ^10^, ^12^ Seracchioli et al found that both cyclic and continuous OC use effectively reduces the size of endometrioma at the time of recurrence and delays endometrioma recurrence.^13^ Other research suggested that instead of producing cytoreductive effects, hormonal agents simply suppress cell proliferation and induce a cytostatic state in the ectopic endometrium as long as they are continued.^5^ OC use, particularly extended or continuous use, can control not only the pain associated with endometriosis but also the progression of lesion in various endometriosis subtypes.

Despite potential advantages, however, cyclic and extended OC regimens carry the risk of side effects that can lead to decreased compliance or to serious deep vein thrombosis. Cyclic regimens are frequently associated with hormone withdrawal symptoms such as headache, bloating, nausea, and breast tenderness during the hormone‐free intervals.^14^ Extended and continuous OC regimens have been associated with a high‐risk of breakthrough bleeding.^14^ In addition, patients may refuse to continue a regimen due to anxiety associated with the loss of regular periods.^15^ However, some women find the absence of menstrual blood flow and associated relief from unwanted symptoms beneficial.

In the present study, we conducted a placebo‐controlled RCT to evaluate the efficacy and safety of a 28‐day Cyclic regimen and 84‐day Extended regimen of NPC‐16 in patients with dysmenorrhea. The primary end point was the efficacy of the Cyclic and Extended NPC‐16 regimens compared to the Placebo regimen. As a secondary end point, we compared the efficacy of the Cyclic and Extended regimens.

## 2 MATERIALS AND METHODS

### 2.1 Study design

This multicenter, randomized, placebo‐controlled trial was conducted between February 2015 and January 2017. Patients with dysmenorrhea were enrolled from 18 private clinics across Japan.

**FIGURE 1:**
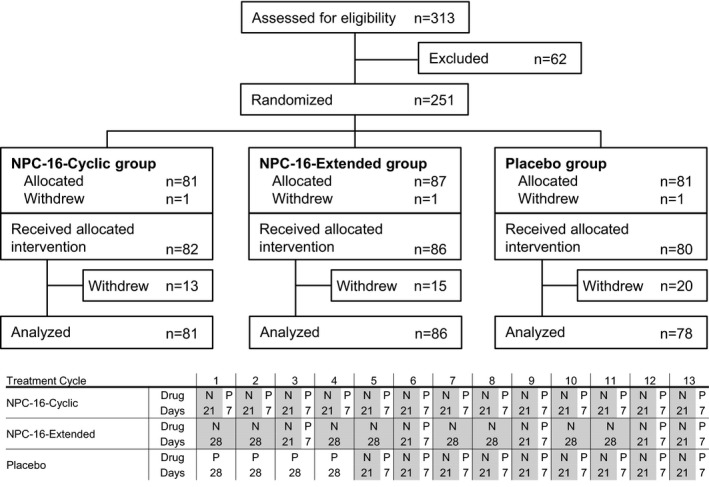
Flow‐chart of patients and study design. *N*, NPC‐16; *P*, Placebo

Treatment started within the first 5 days of each patient's menstrual cycle. Patients allocated to the NPC‐16‐Cyclic regimen (Cyclic group) received NPC‐16 for 21 days, followed by the placebo for 7 days (one cycle: 28 days); this schedule was repeated for 13 cycles. Patients allocated to the NPC‐16‐Extended regimen (Extended group) received NPC‐16 for 77 days, followed by the placebo for 7 days (ie, one 84‐day cycle, equivalent to three 28‐day cycles), and this schedule was repeated for four cycles, followed by one cycle of the Cyclic regimen. After completion of the Extended regimen, an additional cycle of the Cyclic regimen was performed, because safety data for 1‐year (13‐cycle) treatment were required based on a mandate from the authorities. Safety test for drugs was requested to be continued for one year. Patients allocated to the Placebo group received the placebo for 28 days (one cycle); this schedule was repeated for four cycles, followed by nine cycles of the Cyclic regimen (Figure 1). The placebo drugs were administered only four cycles due to the ethical reason for the placebo group.

NPC‐16 (Cyclic and Extended regimens) and the placebo were prepared by the manufacturer (Nobelpharma Co., Ltd.) and supplied in 28‐day blister packs of identical appearance. Use of non‐hormonal agents for analgesic purposes was allowed, at the patient's discretion. However, the use of hormonal drugs other than the trial drug was prohibited. Throughout the term of the study, the use of reliable contraception other than hormonal agents was required for all patients.

Vital signs (blood pressure and body weight) were measured for each patient in each cycle. Clinical laboratory tests, including hematology, blood biochemistry, coagulation and urinalysis, and transvaginal ultrasonography were carried out before treatment and at cycles 3, 6, 9, and 13. Uterine bleeding was evaluated based on entries in diaries recorded by patients throughout the study period. The degree of bleeding was assessed on a 5‐point scale as follows: 0: none, 1: minimal (equivalent to spotting), 2: milder than usual menstruation, 3: similar to usual menstruation; and 4: heavier than usual menstruation.

### 2.2 Study population

Of 313 patients screened, 62 were excluded before randomization, and the remaining 251 patients were randomly assigned. Patients were enrolled if they met the following inclusion criteria: (a) age ≥ 16 years; (b) regular menstrual cycles (28 ± 3 days); (c) diagnosis of primary or secondary dysmenorrhea (diagnostic imaging was indispensable for the diagnosis of primary dysmenorrhea, which was diagnosed considering medical history, pelvic examination results, and the findings of two transvaginal ultrasound examinations; secondary dysmenorrhea was diagnosed when laparoscopy, laparotomy, or two transvaginal ultrasound examinations revealed endometriosis, myoma, or adenomyosis; magnetic resonance imaging was not required); and (d) presence of moderate to severe dysmenorrhea (total dysmenorrhea score of 3 to 6; see Efficacy in the Study Evaluation subsection). Patients who had received medical or surgical treatment for dysmenorrhea within 8 weeks of entry into the trial (including the use of hormonal agents such as estrogen‐ or progestin‐containing medications or the concurrent use of medications that affects the metabolism of estrogen‐ or progestin‐containing medications) were excluded.^6^, ^7^, ^8^

### 2.3 Determination of sample size

The study was designed to simultaneously compare differences in efficacy between the NPC‐16‐ Cyclic and Extended groups versus the Placebo group. Because the Extended regimen was expected to produce a greater change in total dysmenorrhea score compared with the Cyclic regimen, we assumed that a comparison of the Cyclic and Placebo groups would reveal a significant difference. Based on the results of the placebo‐controlled comparison trial of 0.02 mg EE plus 1.0 mg norethisterone (ie, the Cyclic regimen) in treating dysmenorrhea,^8^ it was calculated 70 patients would be needed per group to compare the NPC‐16‐ Cyclic and Placebo groups, assuming a change in score of −2.1 and −1.2 for the NPC‐16‐ Cyclic and Placebo groups, respectively, with a common standard deviation of 1.7, significance level (α) of 0.025, and power (1−β) of 0.8. Considering premature withdrawals, the sample size was set at 80 patients per group.

### 2.4 Randomization and blinding

Patients were randomized to receive the Cyclic regimen, Extended regimen, or Placebo (ratio, 1:1:1). Randomization was stratified by primary and secondary dysmenorrhea (ratio, 1:1). Randomization was carried out according to the permuted block method by a company engaged by Nobelpharma Co., Ltd. For patients with either primary or secondary dysmenorrhea, one block (representing six allocations; two for the Cyclic regimen, two for the Extended regimen, and two for the Placebo) was prepared and allocated to each of the 18 study sites. Information regarding allocation was restricted to the company that carried out the randomization and only after all data had been collected. Both the patients and physicians were blinded to the group to which each patient had been allocated.

### 2.5 Study evaluation

#### 2.5.1 Efficacy

Patients were requested to visit the hospital before, during, and after treatment (total of 19 visits). Baseline data were collected at the pretreatment visit.

The primary end point was total dysmenorrhea score, calculated as the sum of separate scores for limitation of ability to work or study (pain score) and analgesic requirement (drug score). Scores were obtained using verbal rating scales developed by Harada et al.^6^

For the pain score, patients were asked to assess their menstrual pain based on the number of days it limited their ability to work or study according to the following four‐point rating scales: 0, none; 1, mild pain (some loss of work [or study] efficiency); 2, moderate pain (some need to rest in bed, loss of ability to work [or study]); and 3, severe pain (in bed for more than 1 day).

For the drug score, patients were asked to state the number of days they required analgesic drugs to relieve their pain, using similar four‐point rating scale: 0, none; 1, 1 day; 2, 2 days; and 3, ≥3 days.

The secondary end point was the degree of dysmenorrhea as evaluated by patients using a visual‐analog scale (VAS; range, 0‐100). In cases of absence of menstruation, the total dysmenorrhea score and VAS score were recorded as 0. Pain without menstruation was recorded as pelvic pain.

To evaluate the primary end point, the mean difference in total dysmenorrhea score between the mean score at baseline and the mean score after treatment (mean of cycles 1‐3) was calculated, and comparisons were made between the Cyclic group or the Extended group and the Placebo group.

The change in VAS score from baseline to cycle 3 (mean of cycles 1‐3) was compared between the Cyclic or Extended groups and the Placebo group as the secondary end point.

The following parameters were compared between the Cyclic and Extended groups in evaluating the secondary end point: change in total dysmenorrhea score from baseline to each 3‐cycle (mean of cycles 1‐3, 4‐6, 7‐9, and 10‐12), change in VAS of dysmenorrhea and non‐menstrual pelvic pain from baseline to each 3‐cycle interval (mean of cycles 1‐3, 4‐6, 7‐9, and 10‐12), and number of days with uterine bleeding (excluding spotting) at each 3‐cycle interval (mean of cycles 1‐3, 4‐6, 7‐9, and 10‐12).

#### 2.5.2 Adverse effects

Adverse events (AEs) were defined as any unfavorable or unintended clinical signs (including abnormal laboratory values), symptoms or diseases. ADRs were defined as AEs considered related to administration of the study drug.

### 2.6 Statistical methods

Efficacy was evaluated using the full analysis set. The primary end point was compared between the Cyclic group versus the Placebo group and the Extended group versus the Placebo group using change in total dysmenorrhea score from baseline to cycle 3 (cycle 1‐3 mean). To estimate the mean change of each treatment groups, the mixed effect model was performed by using the correlational structure of the mixed model, in which compound symmetry is assumed. This mixed model included interactions between treatment groups and observation cycles as fixed effects and patients as random effects. The multiplicity of two tests was adjusted using simulation methods, as whole alpha errors <5% were considered indicative of statistical significance. For the key secondary end point, VAS was also analyzed using same method used for the primary end point.

Two‐sample t‐tests were performed to compare the following secondary end points between the Cyclic and Extended groups: change in total dysmenorrhea score from baseline, change in VAS score from baseline, and number of days of uterine bleeding (excluding spotting). Multiplicity of tests for 4 terms by 3 cycles (weeks 1‐12, 13‐24, 25‐36 and 37‐48) was adjusted using Bonferroni's method.

Fisher's exact test was used to compare reported or observed AEs and ADRs between the groups. For vital signs and clinical laboratory data, the mean change from baseline at each measurement point after treatment was analyzed using the one‐sample Wilcoxon's signed‐rank test.

## 3 RESULTS

### 3.1 Demographic characteristics and disposition

The median number of patients enrolled from each institution was 11.5 (range, 6‐36; 25th‐75th percentile interquartile range, 9.25‐18).

Figure 1 summarizes the post‐screening patient disposition. Of 313 patients screened, 62 were excluded before randomization, and the remaining 251 were randomly assigned to the Cyclic group (n = 83), Extended group (n = 87), or Placebo group (n = 81). One patient from each group withdrew from the study before administration of the trial drug; therefore, 82, 86, and 80 patients in the Cyclic, Extended, and Placebo groups, respectively, received the trial drug. Baseline demographic characteristics were similar between groups (Table 1).

**TABLE 1:** Baseline patient characteristics

	NPC‐16‐Cyclic	NPC‐16‐Extended	Placebo	*P* value
n	81	86	78	
Age, y	32.1 (8.25)	31.6 (8.80)	33.1 (7.70)	.501*
Weight, kg	54.6 (7.49)	54.2 (8.11)	53.0 (6.12)	.211*
Body mass index, kg/m^2^	21.5 (3.05)	21.4 (3.01)	21.0 (2.62)	.316*
Parity, n (%)				
Nulliparous	53 (65.4)	54 (62.8)	44 (56.4)	.486**
Parous	28 (34.6)	32 (37.2)	34 (43.6)	
Age at menarche, y	12.0 (1.32)	12.1 (1.23)	12.0 (1.25)	.861**
Age at first menstrual pain, y	17.6 (6.18)	17.2 (6.78)	18.1 (6.16)	.694**
Type of dysmenorrhea, n (%)				
Primary	41 (50.6)	44 (51.2)	38 (48.7)	.948**
Secondary	40 (49.4)	42 (48.8)	40 (51.3)	
Endometriosis	29 (72.5)	32 (76.2)	22(55.0)	
Adenomyosis	22 (55.0)	19 (45.2)	23 (57.5)	
Uterine myoma	8 (20.0)	11 (26.2)	10 (25.0)	
Dysmenorrhea score (baseline), n (%)				
3 or 4	48 (59.3)	51 (59.3)	48 (61.5)	.945**
5 or 6	33 (40.7)	35 (40.7)	30 (38.5)	

Note

Values are presented as the mean (±SD) unless otherwise noted.

* Comparison of the three groups by one‐way analysis of variance.

** Comparison of the three groups using the Chi‐squared test

A total of 48 patients (Cyclic group, 13; Extended group, 15; Placebo group, 20) discontinued use of the allocated tablets for the following reasons: in the Cyclic group, patient's request (n = 5), AEs (n = 1), lost to follow‐up (n = 1), conflict with the exclusion criteria (n = 1), worsening of dysmenorrhea (n = 1), pregnancy (n = 2), and physician's discretion (n = 2); in the Extended group, patient's request (n = 5), AEs (n = 7), lost to follow‐up (n = 2), and conflict with the exclusion criteria (n = 1); and in the Placebo group, patient's request (n = 10), AEs (n = 5), lost to follow‐up (n = 1), conflict with the exclusion criteria (n = 1), pregnancy (n = 1), concomitant therapy violation (n = 1), and physician's discretion (n = 1).

### 3.2 Efficacy

One patient from the Cyclic group and two patients from the Placebo group were excluded from the efficacy analysis population because their efficacy data were not collected. Table 2 shows the change from baseline (difference between baseline and the mean of cycles 1‐3) in the total dysmenorrhea and VAS scores.

**TABLE 2:** Efficacy of treatment: change from baseline (difference between baseline and the mean of cycles 1‐3)

	NPC‐16‐Cyclic	NPC‐16‐Extended	Placebo
Total dysmenorrhea score			
Overall dysmenorrhea	−1.8 (0.12; −2.1, −1.6)^a^	−3.1 (0.12; −3.3, −2.8)^b^	−0.9 (0.13; −1.1, −0.6)
Primary dysmenorrhea	−1.9 (0.18; −2.3, −1.6)^a^	−2.8 (0.17; −3.2, −2.5)^b^	−0.8 (0.18; −1.2, −0.5)
Secondary dysmenorrhea	−1.7 (0.18; −2.0, −1.3)^a^	−3.3 (0.17; −3.7, −3.0)^b^	−0.9 (0.18; −1.3, −0.6)
Total dysmenorrhea score			
3 or 4 at baseline	−1.8 (0.15; −2.1, −1.5)^a^	−2.6 (0.14; −2.9, −2.3)^b^	−0.6 (0.15; −0.9, −0.4)
5 or 6 at baseline	−1.9 (0.20; −2.3, −1.5)^a^	−3.8 (0.19; −4.2, −3.4)^b^	−1.2 (0.20; −1.6, −0.8)
Visual analog scale score	−22.6 (1.93; −26.4, −18.9)^a^	−39.7 (1.89; −43.4, −36.0)^b^	−9.5 (1.97; −13.4, −5.6)

Note

Values are presented as the least‐squares (LS) mean (± standard error [SE]; 95% confidence interval).

LS means and SEs of the efficacy variables were calculated using a mixed model for repeated measures.

^a^ *P* < .01 Adjusted *P*‐values obtained by simulation‐based multiple comparisons. NPC‐16‐ Cyclic versus. Placebo.

^b^ *P* < .01 Adjusted *P*‐values obtained by simulation‐based multiple comparisons. NPC‐16‐ Extended versus. Placebo.

#### 3.2.1 Total dysmenorrhea score

Overall, the reduction in total dysmenorrhea score was significantly greater in both the Cyclic group (–1.8) and Extended group (–3.1) than in the Placebo group (–0.9; *P*<.01) among patients with dysmenorrhea (Table 2).

In patients with primary and secondary dysmenorrhea, the respective reductions in total dysmenorrhea score were significantly greater in the Cyclic group (−1.9, −1.7) and Extended group (−2.8, −3.3) than in the Placebo group (−0.8, −0.9; *P*<.01, *P*<.01; Table 2).

**FIGURE 2:**
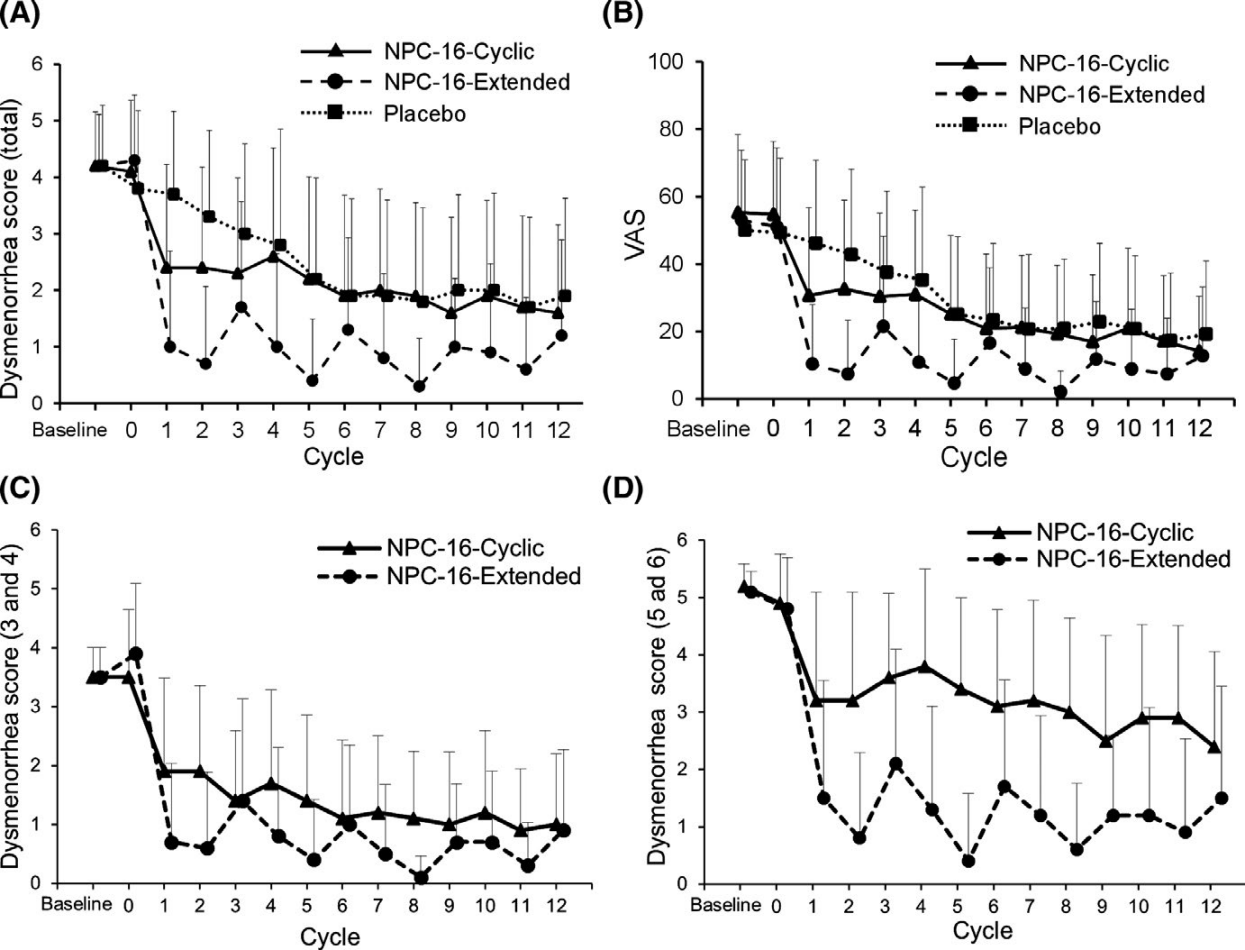
Change in total dysmenorrhea and visual analog scale scores. Change in total dysmenorrhea score (A) and visual analog scale score (B) among all patients, and change in total dysmenorrhea score in patients with a baseline total dysmenorrhea score of 3 or 4 (C) or those with a baseline total dysmenorrhea score of 5 or 6 (D) for the Cyclic versus. Extended regimens. VAS, visual analog scale

As shown in Figure 2A, the reduction in total dysmenorrhea score was greater in the Extended group than Cyclic and Placebo group. Changes in total dysmenorrhea score in the Cyclic and Extended groups according to severity of dysmenorrhea at baseline as shown in Figure 2C,D. In patients with moderate dysmenorrhea (total dysmenorrhea score, 3 or 4), the reduction in total dysmenorrhea score was greater in the Extended group (−2.6) than the Cyclic group (−1.8). In patients with severe dysmenorrhea (total dysmenorrhea score, 5 or 6), the reduction in total dysmenorrhea score was more notable in the Extended group (−3.8) than in the Cyclic group (−1.9; Table 2).

**FIGURE 3:**
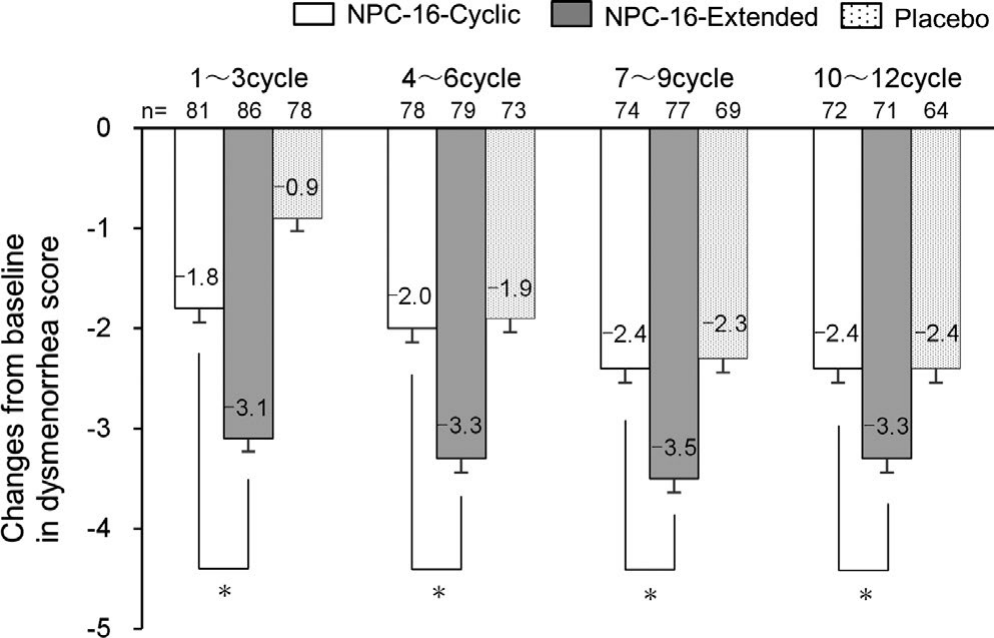
Change in dysmenorrhea score from baseline in the Cyclic and Extended groups. Least‐squares means were calculated using a mixed model for repeated measures for change in the dysmenorrhea score from baseline to the mean of 3 cycles. ^*^ *P* < .001 by two‐sample *t*‐test for NPC‐16‐Cyclic versus NPC‐16‐ Extended adjusted using Bonferroni's method

We also directly compared changes in dysmenorrhea score from baseline between the Cyclic and Extended groups. As clearly illustrated in Figure 3, the dysmenorrhea scores for each respective 3‐cycle interval were significantly reduced in the Extended group (−3.1, −3.3, −3.5, −3.3) as compared to the Cyclic group (−1.8, −2.0, −2.4, −2.4; *P*<.001 for all).

#### 3.2.2 VAS score

Overall, the reduction in VAS score among patients with dysmenorrhea was significantly greater in the Cyclic group (–22.6) and Extended group (–39.7) than the Placebo group (–9.5; *P*<.01; Table 2). As shown in Figure 2B, the reduction in VAS score was greater in the Extended group than the Cyclic group. The VAS score for non‐menstrual pelvic pain was significantly reduced from baseline in both the Cyclic and Extended groups (data not shown).

### 3.3 Safety

#### 3.3.1 ADRs during cycles 1‐3

The incidence of ADRs was 67.1% in the Cyclic group and 97.7% in the Extended group, in each group significantly greater than in the Placebo group (50.0%). The incidence of irregular uterine bleeding was significantly greater in the Cyclic group (50.0%) than the Placebo group (25.0%). In the Extended group, the incidences of lower abdominal pain, amenorrhea, oligomenorrhea, polymenorrhea, and irregular uterine bleeding (22.1, 8.1, 59.3, 17.4, and 90.7%, respectively) were significantly higher than in the Placebo group (8.8, 0.0, 3.8, 1.3, and 25.0%, respectively; data not shown).

#### 3.3.2 Safety throughout the study period

The incidence of ADRs was significantly higher in the Extended group (98.8%) than in Cyclic group (84.1%). In the Extended group, the incidences of lower abdominal pain, amenorrhea, oligomenorrhea, and irregular uterine bleeding (31.4, 31.4, 86.0, and 95.3%, respectively) were significantly higher than in the Cyclic group (12.2, 1.2, 24.4, and 72.0%, respectively).

No severe ADRs were reported in any of the groups. No clinically significant changes in laboratory test values and vital signs were recorded in either the Cyclic or Extended group.

Changes in the incidence of non‐menstrual abnormal uterine bleeding (not spotting) in the Cyclic and Extended groups are summarized in Table 3. The number of days of abnormal uterine bleeding during each 3‐cycle interval was greater in the Extended group than the Cyclic group (*P* < .001 for all) through 12 cycles. Although the number of days of irregular uterine bleeding in the Extended group gradually declined, it was still significantly greater than that of the Cyclic group.

**TABLE 3:** Numbers of days with uterine bleeding (excluding spotting; difference between baseline and the mean of 3 cycles)

	NPC‐16‐Cyclic	NPC‐16‐Extended	*P* value*
Numbers of days with uterine bleeding (excluding spotting)			
Cycles 1‐3	5.4 (0.86)	16.7 (0.83)	<.01
Cycles 4‐6	2.9 (0.87)	10.8 (0.86)	<.01
Cycles 7‐9	2.8 (0.88)	9.0 (0.86)	<.01
Cycles 10‐12	3.1 (0.89)	8.3 (0.88)	<.01

Note

Values are presented as the least squares (LS) mean (± standard error [SE]).

LS means were calculated using a mixed model for repeated measures of the mean of 3 cycles in days with vaginal bleeding.

* *P* < .01 by two‐sample *t*‐test for NPC‐16‐ Extended regimen versusNPC‐16‐ Cyclic regimen adjusted using Bonferroni's.

## 4 DISCUSSION

We conducted the present RCT to evaluate the efficacy of Cyclic and Extended regimens of NPC‐16 (containing EE plus levonorgestrel) compared with Placebo in patients with dysmenorrhea. Compared with Placebo, both the Cyclic and Extended NPC‐16 regimens significantly reduced the total dysmenorrhea and VAS scores. With regard to the secondary end point, the Extended regimen was superior to the Cyclic regimen in alleviating primary and secondary dysmenorrhea.

The present RCT also compared the efficacy of the Cyclic and Extended regimens with respect to the severity of dysmenorrhea. In patients with severe dysmenorrhea at baseline (total dysmenorrhea score, 5 or 6), the Extended regimen produced a more pronounced reduction in the total dysmenorrhea score compared to the Cyclic regimen. Although high‐quality evidence of the effects of OCs on dysmenorrhea is limited, the results of the present study provide further support for the hypothesis that the Extended regimen is superior in the treatment of patients with severe dysmenorrhea involving endometriosis.

Primary and secondary dysmenorrhea patients were treated together in the present study. In modern medical management of endometriosis, hormone treatment is commenced when patients see physicians before a surgical diagnosis of endometriosis is made.^4^ Thus, dysmenorrhea regardless of whether it is primary or secondary is an indication for OC treatment in patients with suspected endometriosis.

Extended or continuous OC regimens were introduced for contraceptive purposes, because the 7‐day hormone‐free interval in cyclic regimens is associated with hormone withdrawal symptoms, including headache and mood swing.^14^ Regular menstruation, however, is also associated with dysmenorrhea and other related symptoms.

Vercellini et al treated endometriosis‐associated pain with 2 years of continuous OCs in 50 women with severe dysmenorrhea in which treatment with cyclic OCs after endometriosis surgery failed.^15^ They reported a statistically significant reduction in the verbal and visual analog pain scores. Most of the patients (84%) were satisfied with the treatment. Moreover, a meta‐analysis revealed significantly lower dysmenorrhea recurrence rates after endometrioma surgery with a continuous OC regimen, although the difference in cyst recurrence was not statistically significant.^16^ These results suggest that a continuous OC regimen should be recommended post‐surgery for patients with dysmenorrhea.

International guidelines describe OCs as the first‐choice treatment option for endometriosis‐associated pelvic pain and dysmenorrhea that is not responsive to pain medications.^17^, ^18^, ^19^ Recent reviews also suggest that low‐dose OCs or low‐cost progestins should be considered as first‐line medications.^4^, ^20^ Vercellini et al suggested that continuous or extended OC regimens should be recommended, although data regarding the efficacy of cyclic and continuous or extended regimens are limited.^20^

In young women, long‐term (spanning years) hormone treatment is necessary to control pain and endometrioma recurrence after surgery.^4^, ^20^ Casper published an interesting opinion paper in which he pointed out a contradiction with regard to the use of OCs for treating endometriosis, as OCs contain both estrogen and progestin.^21^ Low‐dose OCs contain 0.02 mg EE, which is equivalent to 4 mg of micronized estradiol or 4 tablets consisting of 0.625 mg of conjugated equine estrogen. Such high estrogen concentrations in OCs could stimulate the proliferation of endometriosis tissues, although progestins in the OCs antagonize the effect of estrogen on the endometrium and endometriosis tissues. Although Casper suggested that progestins are preferable to OCs as a first‐line treatment, the efficacy of OCs may be underestimated in the literature due to a lack of data regarding non‐menstrual pelvic pain and deep pain, including dyspareunia and dyschezia.^21^, ^22^ Previously and in the present study, we demonstrated that both cyclic and extended OC regimens improve dysmenorrhea and also relieve non‐menstrual pelvic pain and dyspareunia when used long‐term.^10^ Additionally, when progestin is used for many years, hypoestrogenic symptoms, including a reduction in bone mineral density, can be of concern, especially in adolescent and women in their 20s or 30s.^23^, ^24^ It may be continued for years because discontinuation of hormone treatment can be associated with symptom recurrence or re‐growth of lesions.

EE decreases the likelihood of irregular uterine bleeding, an ADR associated with progestin.^25^ However, EE also increases the risk of venous thromboembolism, which although rare is potentially fatal. In the present study, the incidence of irregular uterine bleeding was higher in the Extended group than in the Cyclic group but tended to decrease over time.

Continuous and extended OC regimens are recommended for treating pain in endometriosis patients, but high‐quality background evidence of efficacy is still lacking. The efficacy of low‐dose OC Extended regimen was compared directly with that of the Cyclic regimen in patients with secondary dysmenorrhea, including that caused by endometriosis. The results showed for the first time that the dysmenorrhea scores significantly decreased in the Extended group compared to the Cyclic group. Moreover, the new finding in the present study is that the NPC‐16‐Extended regimen may be more beneficial for patients with severe dysmenorrhea. But it was evaluated as a secondary end point in the present study. This is a weak point of the present study.

The present study confirmed that compared with placebo, administration of the low‐dose OC NPC‐16 as a Cyclic or Extended regimen, is efficacious for treating dysmenorrhea. The Extended regimen was more efficacious in the treatment of severe cases, although it was associated with a higher incidence of irregular uterine bleeding. NPC‐16 is a useful option for the treatment of both primary and secondary dysmenorrhea, including that associated with endometriosis.

## DISCLOSURES

*Conflicts of interest*: Tasuku Harada and Mikio Momoeda received consulting fees from Nobelpharma Co., Ltd.

## ETHICAL APPROVAL

The study protocol (number NPC‐16‐2) was approved by the institutional review board at each study site. Throughout the course of the trial, monitors made regular visits to each study site to ensure adherence to the protocol. All patients provided written informed consent.
